# Response to PEEP in COVID-19 ARDS patients with and without extracorporeal membrane oxygenation. A multicenter case–control computed tomography study

**DOI:** 10.1186/s13054-022-04076-z

**Published:** 2022-07-02

**Authors:** Jean-Christophe Richard, Florian Sigaud, Maxime Gaillet, Maciej Orkisz, Sam Bayat, Emmanuel Roux, Touria Ahaouari, Eduardo Davila, Loic Boussel, Gilbert Ferretti, Hodane Yonis, Mehdi Mezidi, William Danjou, Alwin Bazzani, Francois Dhelft, Laure Folliet, Mehdi Girard, Matteo Pozzi, Nicolas Terzi, Laurent Bitker

**Affiliations:** 1 Service de Médecine Intensive Réanimation, Hôpital de la Croix Rousse, Hospices Civils de Lyon, 103 Grande Rue de la Croix Rousse, 69004 Lyon, France; 2 INSA-Lyon, CNRS, INSERM, CREATIS UMR 5220, Univ Lyon, Université Claude Bernard Lyon 1, U1294 Villeurbanne, France; 3 Université de Lyon, Université Claude Bernard Lyon 1, Villeurbanne, France; 4 Service de Médecine-Intensive Réanimation, CHU Grenoble-Alpes, Grenoble, France; 5 Synchrotron Radiation for Biomedicine Laboratory (STROBE), INSERM UA07, Univ. Grenoble Alpes, Grenoble, France; 6 Department of Pulmonology and Physiology, Grenoble University Hospital, Grenoble, France; 7 Service de Radiologie, Hôpital De La Croix Rousse, Hospices Civils de Lyon, Lyon, France; 8 Grenoble, France Service de Radiologie Diagnostique Et Interventionnelle, Université Grenoble Alpes, CHU Grenoble-Alpes, Grenoble, France; 9 Service de Chirurgie Cardiaque, Hôpital Louis Pradel, Hospices Civils de Lyon, Lyon, France; 10 INSERM, U1042, University Grenoble Alpes, HP2 Grenoble, France

**Keywords:** ARDS, Computed tomography, ECMO, PEEP, COVID-19

## Abstract

**Background:**

PEEP selection in severe COVID-19 patients under extracorporeal membrane oxygenation (ECMO) is challenging as no study has assessed the alveolar recruitability in this setting. The aim of the study was to compare lung recruitability and the impact of PEEP on lung aeration in moderate and severe ARDS patients with or without ECMO, using computed tomography (CT).

**Methods:**

We conducted a two-center prospective observational case–control study in adult COVID-19-related patients who had an indication for CT within 72 h of ARDS onset in non-ECMO patients or within 72  h after ECMO onset. Ninety-nine patients were included, of whom 24 had severe ARDS under ECMO, 59 severe ARDS without ECMO and 16 moderate ARDS.

**Results:**

Non-inflated lung at PEEP 5 cmH_2_O was significantly greater in ECMO than in non-ECMO patients. Recruitment induced by increasing PEEP from 5 to 15 cmH_2_O was not significantly different between ECMO and non-ECMO patients, while PEEP-induced hyperinflation was significantly lower in the ECMO group and virtually nonexistent. The median [IQR] fraction of recruitable lung mass between PEEP 5 and 15 cmH_2_O was 6 [4–10]%. Total superimposed pressure at PEEP 5 cmH_2_O was significantly higher in ECMO patients and amounted to 12 [11–13] cmH_2_O. The hyperinflation-to-recruitment ratio (i.e., a trade-off index of the adverse effects and benefits of PEEP) was significantly lower in ECMO patients and was lower than one in 23 (96%) ECMO patients, 41 (69%) severe non-ECMO patients and 8 (50%) moderate ARDS patients. Compliance of the aerated lung at PEEP 5 cmH_2_O corrected for PEEP-induced recruitment (C_BABY LUNG_) was significantly lower in ECMO patients than in non-ECMO patients and was linearly related to the logarithm of the hyperinflation-to-recruitment ratio.

**Conclusions:**

Lung recruitability of COVID-19 pneumonia is not significantly different between ECMO and non-ECMO patients, with substantial interindividual variations. The balance between hyperinflation and recruitment induced by PEEP increase from 5 to 15 cmH_2_O appears favorable in virtually all ECMO patients, while this PEEP level is required to counteract compressive forces leading to lung collapse. C_BABY LUNG_ is significantly lower in ECMO patients, independently of lung recruitability.

**Supplementary Information:**

The online version contains supplementary material available at 10.1186/s13054-022-04076-z.

## Background

Lung recruitability of COVID-19 acute respiratory distress syndrome (ARDS) remains to date a matter of debate, as conflicting results have been reported using several techniques derived from change in respiratory mechanics and/or gas exchange in response to positive end-expiratory pressure (PEEP) increase [1–5]. Various factors may explain the heterogeneity of literature reports regarding recruitability of COVID-19 ARDS: time from ARDS onset, ARDS severity, reliability of these methods to compute recruitment, ventilatory management before evaluation, body mass index (BMI), among others. Furthermore, COVID-19 ARDS lungs may be intrinsically less recruitable than “classical” ARDS, as non-aerated lung regions in COVID-19 patients may represent alveolar spaces substituted by fibrosis, cellular debris and necrotic tissue rather than atelectasis [6].

Two small studies have been published so far using computed tomography (CT) in the context of COVID-19 ARDS and provided conflicting results [7, 8]. One study performed during the later phase of the disease identified most patients as being non-recruiters by PEEP [7], while median recruitability in the other study [8] performed within 72  h of ARDS onset was substantially higher than in non-COVID ARDS historical studies [9].

No study has assessed to date the recruitability of severe COVID-19 ARDS under veno-venous extracorporeal membrane oxygenation (ECMO). In non-COVID-19 ARDS patients, median lung recruitability was substantially higher in 47 patients under ECMO [10] than in the seminal study of Gattinoni performed on non-ECMO ARDS patients [9], suggesting that higher PEEP levels should be beneficial in ARDS patients under ECMO. Whether these results apply to COVID-19 ARDS patients under ECMO remains to date unknown, as well as optimal PEEP level in this subset of patients. We hypothesized that lung recruitability is influenced by time from ARDS onset and may be lower in ECMO patients cannulated more than 72  h after ARDS onset. Furthermore, we hypothesized that compliance of the baby lung (i.e., aerated lung at PEEP 5 cmH_2_O) may explain poor lung recruitability, as it may be an early marker of fibrosis.

The primary aim of the study was to compare lung recruitability assessed with CT in moderate and severe ARDS patients with or without ECMO. The secondary aim of the study was to identify the mechanisms underlying lung recruitability in COVID-19 ARDS.

## Methods

### Study design and setting

The study is a multicenter prospective observational case–control study, performed in 2 intensive care units located in university hospitals, and was conducted in accordance with the amended declaration of Helsinki. The study was approved by a local independent ethics committee (Comité Scientifique et Ethique des Hospices Civils de Lyon, 20_194) and complied to the STROBE criteria for observational studies [11]. Patients were enrolled between November 1, 2020, and June 16, 2021, in center#1 and between November 1, 2020, and December 31, 2021, in center#2. Consent for data utilization was sought from the patients or their representative, and follow-up lasted 90 days. The primary endpoint of the study was the amount of recruitable lung between PEEP 5 and 15 cmH_2_O on CT.

### Patients and protocol

Eligible participants were ARDS patients [12] aged 18 years or older, under invasive mechanical ventilation, who had a COVID-19 pneumonia with a positive SARS-CoV-2 reverse transcription polymerase chain reaction, and an indication for CT.

Exclusion criteria were ARDS onset > 72 h in non-ECMO patients, ECMO onset > 72 h, contra-indication to transport to the imaging facility (ratio of oxygen arterial partial pressure to inspired oxygen fraction (PaO_2_/FiO_2_) < 60 Torr, mean arterial pressure < 65 mmHg, or intracranial hypertension), chronic obstructive pulmonary disease, pneumothorax or bronchopleural fistula, previous inclusion in the present study, the presence of intrathoracic metallic devices, pregnancy, patient under a legal protective measure, and refusal to participate by patient and/or relative.

Non-ECMO patients were ventilated with a tidal volume (VT) of 4 to 6 mL.kg^−1^ of predicted body weight (PBW) to keep plateau pressure (*P*_Plat,rs_) below 30 cmH_2_O, with recommendation to use a PEEP-FiO_2_ table to adjust PEEP [13]. ECMO patients were ventilated with a VT of 1 mL.kg^−1^ PBW, with PEEP adjusted to target a P_Plat,rs_ approximating 20–22 cmH_2_O.

Respiratory measurements and arterial blood gas analysis were performed at least 1 h after adjustment of ventilatory settings. Patients were then transferred to the imaging facility using a transport ventilator (MONNAL T60—Air Liquide Medical Systems, Antony, France) with unchanged ventilatory settings. The endotracheal tube was transiently occluded with a Kocher clamp during ventilator change to avoid derecruitment.

### Data analysis

Total PEEP (PEEP_tot,rs_) and P_Plat,rs_ were measured at the end of 3-s end-expiratory and end-inspiratory pauses. Airway driving pressure (ΔP_rs_) was computed as P_Plat,rs_ minus PEEP_tot,rs_. Elastance of the respiratory system was computed as Δ*P*_rs_ divided by VT.

Low-dose CT acquisitions were performed in the supine position with an iCT 256 Ingenuity CT (Philips Healthcare, Eindhoven, The Netherlands), or a GE Optima CT scan (GE Medical Systems, Milwaukee, USA) using the following settings: voltage 140 kVP, slice thickness 1 mm, and matrix size 512 × 512. Field of view, pixel size, and mAs were adapted for each patient. Lung scanning was performed from apex to base during end-expiratory and end-inspiratory pauses at the PEEP level set by the clinician (CT_Expi-Inspi_), and during end-expiratory pauses at PEEP 15 and 5 cmH_2_O (CT_PEEP5-15_). Lack of respiratory efforts during the pauses was visually checked on the ventilator pressure–time curves. Image reconstruction was performed using a smooth filter (kernel B). The lungs were interactively segmented with a CreaTools-based software [14], excluding pleural effusions, hilar and mediastinal structures. Segmented lung volumes were analyzed using MATLAB (MathWorks, Natick, MA).

Voxel tissue and gas fraction were computed as previously described [15]. Tissue and gas volumes were computed as the product of their respective fractions times voxel volume times number of voxels in segmented lung volume, respectively.

Lung parenchyma was classified into four compartments, according to CT number: non-inflated (density between + 100 and − 100 Hounsfield units (HU)), poorly inflated (density between − 101 and − 500 HU), normally inflated (density between − 501 and − 900 HU), and hyperinflated tissue (density ≤ − 901 HU).

Total lung weight and weight of each compartment were estimated using lung tissue volume, assuming a tissue density of 1 g.mL^−1^ [16].

VT was assessed on CT (VT_CT_) by subtracting the volume of gas at end-inspiration and at end-expiration in segmented lungs.

The amount of recruitable lung between PEEP 5 and 15 cmH_2_O (∆PEEP_5-15_-induced recruitment) was computed as the weight of the non-inflated compartment at PEEP 5 cmH_2_O minus its weight at PEEP 15 cmH_2_O, and standardized to total lung weight.

Tidal recruitment of the non-inflated compartment was defined as the weight of the non-inflated compartment at end-expiration minus its weight at end-inspiration [17], and standardized to total lung weight.

Change in lung aerated volume induced by PEEP change from 5 to 15 cmH_2_O (PEEP_volume_) was computed as the difference in the total volume of gas within the lungs between PEEP 15 and 5 cmH_2_O.

The hyperinflation-to-recruitment ratio was computed as the difference between hyperinflated compartment total volume at PEEP 15 minus its value at PEEP 5 cmH_2_O, over the difference between non-inflated compartment total volume at PEEP 5 minus its value at PEEP 15 cmH_2_O [8].

Tidal hyperinflation was computed as the volume of the hyperinflated compartment at end-inspiration minus its volume at end-expiration [17], and standardized to predicted body weight.

The total superimposed pressure in the most dorsal parts of the lung was computed as previously described [18].

The lung inhomogeneity extent was measured by comparing the inflation of neighboring lung regions as previously described [19, 20] and was defined as the percentage of lung volume presenting an inflation ratio of neighboring regions greater than 1.61 (i.e., the 95th percentile of a control population) [19].

We finally developed a method to estimate elastic properties of the already aerated lung at PEEP 5 cmH_2_O with CT (C_BABY LUNG_, Additional file 1). Classical computation of compliance between PEEP 5 and 15 cmH_2_O (i.e., change in lung aerated volume divided by change in PEEP) overestimates C_BABY LUNG_ as recruited alveoli account partly for the change in aeration. As recruitment assessed by CT is computed as the difference in non-aerated lung compartment weight between PEEP levels, a computation of recruited aerated volume (Rec_Aer vol_) from recruited lung weight was performed using the methodology proposed by Paula and coworkers [21], assuming that recruitable alveoli would remain aerated at PEEP 5 cmH_2_O and have equilibrated to a level of expansion equivalent to that of other already open alveoli at PEEP 5 [21]. C_BABY LUNG_ between PEEP 5 and 15 cmH_2_O was finally computed as: (PEEP_volume_—Rec_Aer vol_)/∆PEEP (i.e., 10 cmH_2_O).

A quality control was performed on both couples of CT images (CT_Expi-Inspi_, CT_PEEP5-15_). Images couples with segmented lung weight differing by more than 5% were excluded. CT_Expi-Inspi_, in which VT_CT_ differed from VT set on the ventilator by more than 60 mL were also excluded (Additional file 2).

### Statistical analysis

Statistical analysis was performed using R version 4.1.1 [22] with packages multcomp [23], lme4 [24], lmerTest [25], and interactions [26]. A *p* value ≤ 0.05 was chosen for statistical significance.

Data were expressed as count (percentage) or median [interquartile range] and compared between groups with the Fisher’s exact test for categorical variables and ANOVA for continuous variables. Multiple comparisons between groups were made using the Holm–Sidak procedure. Comparisons of variables involving the same individual were made with linear mixed models, using patient as a random effect.

Multivariate analyses were performed using linear models, by incorporating variables with *p* values < 0.2 in univariate analysis and stepwise backward selection.

Estimation of sample size was not computed as the study is exploratory, and data collection stopped with the control of COVID-19 fifth wave in our geographic area.

Missing data were not imputed owing to the low rate of missingness for variables included in the multivariate models (Additional file 3).

## Results

Flowchart of the study is presented in Additional file 2. Ninety-nine patients were included, of whom 24 were severe ARDS under ECMO, 59 severe ARDS without ECMO and 16 moderate ARDS. Three non-ECMO patients with delay between ARDS onset > 72 h (4 to 6 days after ARDS onset) were erroneously included and remained in the analysis. Patients’ characteristics at inclusion are reported in Table 1, and respiratory mechanics and blood gas data are presented in Table 2.

**Table 1:** Patient characteristics

Variables	Whole dataset (*n* = 99)	Moderate ARDS without ECMO (*n* = 16)	Severe ARDS without ECMO (*n* = 59)	Severe ARDS on ECMO (*n* = 24)
Sex male—no. (%)	75 (76%)	14 (88%)	42 (71%)	19 (79%)
Age—yr	62 [54–71]	61 [55–70]	66 [57–73]	57 [50–61]^b^
BMI—kg.m^−2^	30 [26–37]	29 [25–32]	30 [26–37]	32 [27–39]
Delay between hospital admission and CT—day	5 [3–8]	5 [3–6]	4 [3–7]	10 [6–12] ^b^
Delay between ICU admission and CT—day	3 [1–6]	3 [1–5]	2 [1–3]	7 [5–8]^a,b^
Delay between ARDS onset and CT—day	1 [1–3]	1 [1, 2]	1 [1, 2]	5 [3–7] ^a,b^
Delay between IMV onset and CT—day	1 [1–3]	1 [1, 2]	1 [1, 2]	5 [3–7] ^a,b^
SAPS 2 at ICU admission	39 [30–45]	41 [28–45]	40 [31–47]	36 [30–42]
SOFA score at inclusion	7 [5–8]	6 [5–7]	7 [4–8]	7 [5–9]
Prone position in the 24 h preceding CT—no. (%)	81 (82%)	14 (88%)	46 (78%)	21 (88%)
iNO in the 24 h preceding CT—no. (%)	19 (19%)	0 (0%)	12 (20%)	7 (29%)
NMBA in the 24 h preceding CT—no. (%)	96 (96%)	15 (94%)	57 (97%)	24 (100%)
RRT in the 24 h preceding CT—no. (%)	4 (4%)	1 (6%)	2 (3%)	1 (4%)
Inotropes in the 24 h preceding CT—no. (%)	1 (1%)	0 (0%)	1 (2%)	0 (0%)
Vasopressor in the 24 h preceding CT—no. (%)	67 (68%)	13 (81%)	37 (63%)	17 (71%)
ICU mortality—no. (%) ^c^	49 (49%)	3 (19%)	32 (54%)	14 (58%)
Day-90 mortality—no. (%) ^c^	48 (49%)	3 (19%)	32 (55%)	13 (54%)
Ventilator-free days at day 90—day	0 [0–71]	59 [13–83]	0 [0–70]	0 [0–56]
ICU length of stay—day	25 [16–40]	24 [13–45]	23 [16–36]	36 [21–48]
Hospital length of stay—day	37 [23–58]	44 [28–78]	35 [21–49]	41 [29–59]

Values are median [1st quartile–3rd quartile] or count (percentage)

^a^ *p* < 0.05 vs. moderate ARDS without ECMO. ^b^ *p* < 0.05 vs. severe ARDS without ECMO, ^c^ *p* < 0.05 between groups

*ARDS* acute respiratory distress syndrome, *BMI* body mass index, *CT* computed tomography, *ECMO* extracorporeal membrane oxygenation, *ICU* intensive care unit, *IMV* invasive mechanical ventilation, *iNO* inhaled nitric oxide, *NMBA* neuromuscular blocking agents, no number, *RRT* renal replacement therapy, *SAPS2* Simplified Acute Physiology Score

**Table 2:** Respiratory mechanics and arterial blood gas

Variables	Whole dataset (*n* = 99)	Moderate ARDS without ECMO (*n* = 16)	Severe ARDS without ECMO (*n* = 59)	Severe ARDS on ECMO (*n* = 24)
PEEP—cmH_2_O	10 [5–14]	9 [5–10]	10 [5–10]	15 [13–15] ^a,b^
VT—mL.kg^−1^ PBW	5.9 [3.9–6.0]	6.0 [5.9–6.0]	6.0 [5.9–6.0]	1.0 [1.0–1.0] ^a,b^
RR—min^−1^	22 [16–28]	23 [20–28]	25 [22–29]	5 [5–10] ^a,b^
PEEP_tot,rs_—cmH_2_O	10 [7–15]	9 [6–10]	10 [6–11]	15 [14–15] ^a,b^
P_plat,rs_—cmH_2_O	20 [17–22]	18 [17–24]	20 [16–23]	20 [19–21]
P_peak_—cmH_2_O	29 [25–35]	30 [28–34]	32 [27–37]	22 [21–24] ^a,b^
ΔP_rs_—cmH_2_O	9 [7–12]	10 [9–13]	10 [9–12]	5 [3–8] ^a,b^
E_rs_—cmH_2_O. L^−1^	33 [25–44]	29 [22–37]	28 [23–35]	70 [53–95] ^a,b^
pH	7.38 [7.32–7.44]	7.37 [7.33–7.42]	7.37 [7.31–7.41]	7.46 [7.39–7.47] ^a,b^
PaO_2_—Torr	72 [65–80]	78 [67–89]	74 [66–80]	69 [62–74] ^a^
FiO_2_ or FmO_2_ – %	90 [60–100]	50 [44–53]	90 [65–100] ^a^	100 [80–100] ^a^
PaCO_2_—Torr	49 [41–54]	42 [37–51]	50 [43–58]	49 [41–51]
Bicarbonates—mmol.L^−1^	28 [24–32]	26 [22–28]	27 [24–31]	32 [29–34] ^a,b^
Lactate—mmol.L^−1^	1.7 [1.3–2.2]	1.5 [1.3–1.9]	1.6 [1.3–2.2]	1.9 [1.6–2.4]

Values are median [1st quartile–3rd quartile], ^a^ *p* < 0.05 vs. moderate ARDS without ECMO. ^b^ *p* < 0.05 vs. severe ARDS without ECMO

*ARDS* acute respiratory distress syndrome, ΔP_rs_, driving pressure of the respiratory system, *ECMO* extracorporeal membrane oxygenation, *E*_*rs*_ elastance of the respiratory system, *FiO*_*2*_ inspired fraction of oxygen, FmO_2_, *ECMO* membrane oxygen fraction, *PaCO*_*2*_ carbon dioxide partial pressure in arterial blood, *PaO*_*2*_ oxygen partial pressure in arterial blood, *PBW* predicted body weight, *PEEP* positive end-expiratory pressure, *PEEP*_*tot,rs*_ total PEEP of the respiratory system, *P*_*peak*_ peak airway pressure, *P*_*plat,rs*_ plateau pressure of the respiratory system, *RR* respiratory rate, *VT* tidal volume

### Lung CT parameters at PEEP 5 cmH_2_O

**Fig. 1:**
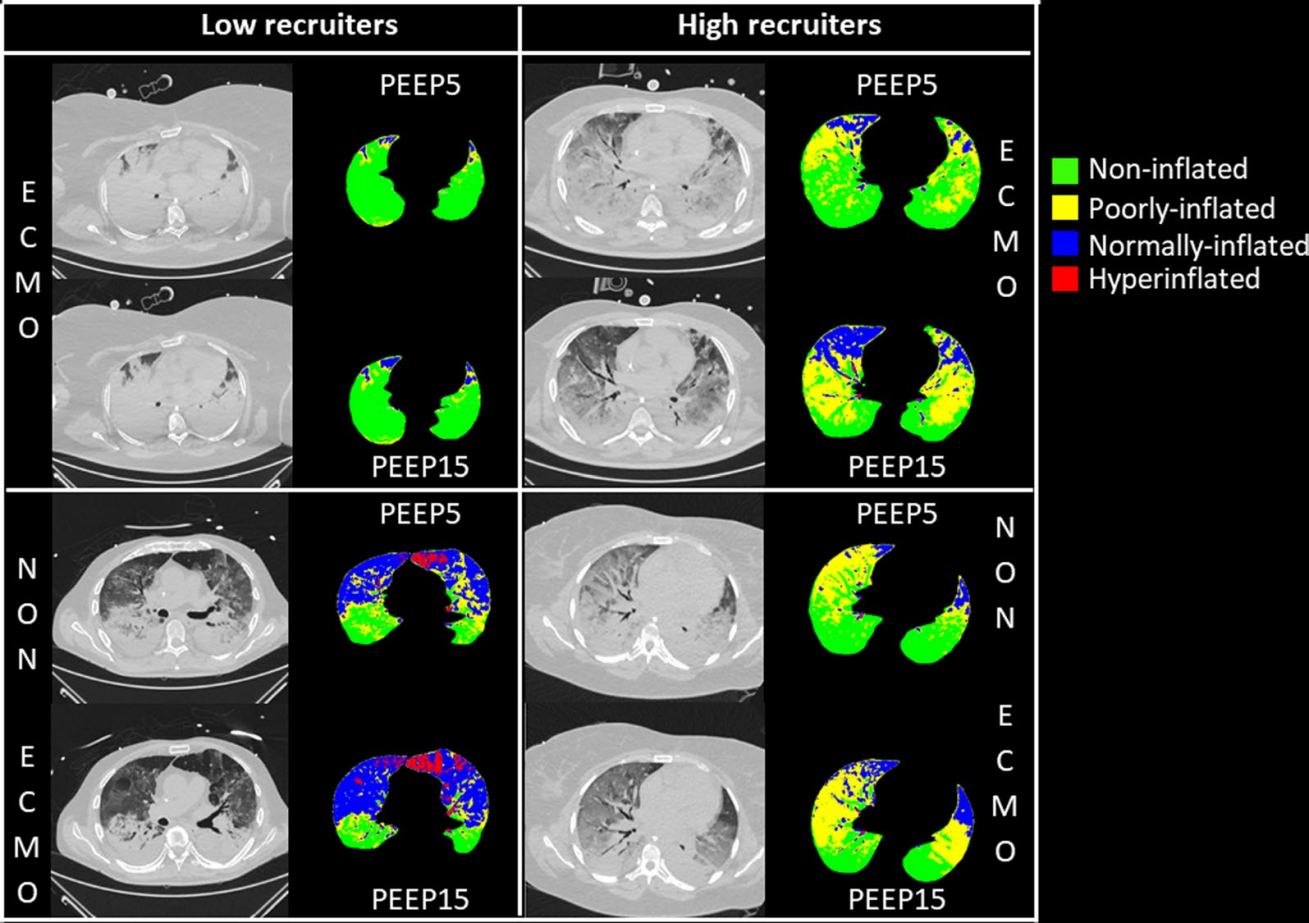
CT images acquired at the mid-chest level in 4 representative patients. ECMO patients are presented in the uppermost quadrants, while non-ECMO patients are presented in the lowermost quadrants. Patients with ∆PEEP_5–15_-induced recruitment below the median value of the whole population (deemed as low recruiters) are presented in the leftmost quadrants, while patients with PEEP_5–15_-induced recruitment above the median value of the whole population (deemed as high recruiters) are presented in the rightmost quadrants. In each quadrant, the upper two images were acquired at PEEP 5 cmH_2_O (one raw CT image on the left and one quantitative parametric CT on the right), and the lower two were acquired at PEEP 15 cmH_2_O. The color code used for parametric images is provided in the figure legend. CT, computed tomography, ∆PEEP_5–15_-induced recruitment, amount of recruitable lung between PEEP 5 and 15 cmH_2_O, ECMO, extracorporeal membrane oxygenation, PEEP, positive end-expiratory pressure

**Fig. 2:**
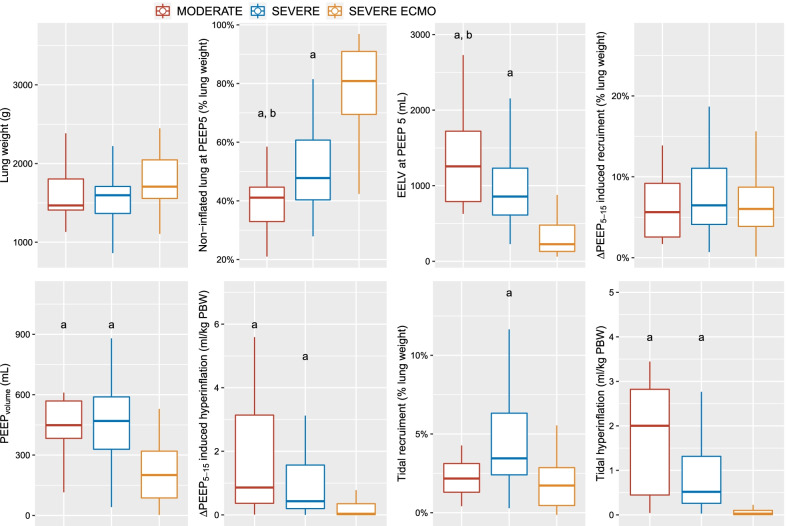
CT parameters as a function of ARDS severity ^a^, *p* < 0.05 vs. severe ARDS with ECMO, ^b^, *p* < 0.05 vs. severe ARDS without ECMO. ARDS, acute respiratory distress syndrome, ∆_PEEP5-15_, change in PEEP from 5 to 15 cmH_2_O, PEEP_volume_, change in lung aeration induced by PEEP change from 5 to 15 cmH_2_O, ECMO, extracorporeal membrane oxygenation, EELV, end-expiratory lung volume, MODERATE, moderate ARDS, PBW, predicted body weight, PEEP, positive end-expiratory pressure, SEVERE, severe ARDS without ECMO, SEVERE ECMO, severe ARDS under ECMO

**Fig. 3:**
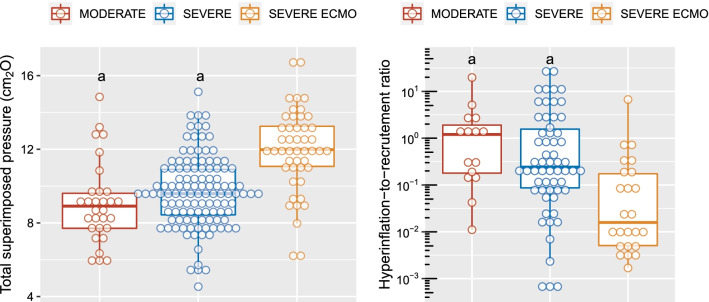
Total superimposed pressure at PEEP 5cmH_2_O and hyperinflation-to-recruitment ratio as a function of ARDS severity. The total superimposed pressure is the hydrostatic pressure superimposed in the most dorsal level of the lungs, computed with CT, assuming that pressure is transmitted through the lung parenchyma as in a fluid. Data points are individual values in each lung for total superimposed pressure and individual values in the whole lung for hyperinflation-to-recruitment ratio. ^a^, *p* < 0.05 vs severe ARDS under ECMO.ARDS, acute respiratory distress syndrome, CT, computed tomography, ECMO, extracorporeal membrane oxygenation, MODERATE, moderate ARDS, PEEP, positive end-expiratory pressure, SEVERE, severe ARDS without ECMO, SEVERE ECMO, severe ARDS under ECMO

CT images obtained in 4 representative ECMO and non-ECMO patients are provided in Fig. 1. Lung weight assessed on CT was not significantly different between groups (Fig. 2). Non-inflated lung at PEEP 5 cmH_2_O was significantly greater in ECMO than in non-ECMO patients (Fig. 2), and in severe ARDS non-ECMO patients vs. moderate ARDS patients. End-expiratory aerated lung volume (EELV) at PEEP 5 cmH_2_O was significantly lower in ECMO patients. Total superimposed pressure at PEEP 5 cmH_2_O (Fig. 3, left panel) was significantly higher in ECMO patients (12 [11-13] cmH_2_O), as compared to both moderate (10 [8-11] cmH_2_O) and severe non-ECMO patients (9 [7-13] cmH_2_O).

### Lung recruitability

**Fig. 4:**
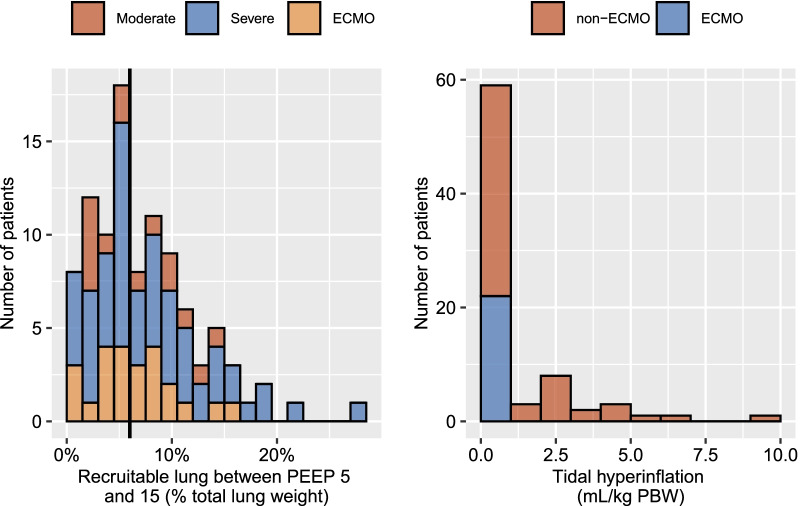
Distribution of patients according to recruitability between PEEP 5 and 15 cmH_2_O or tidal hyperinflation. Vertical bar is the median amount of recruitable lung. ECMO, severe acute respiratory distress syndrome (ARDS) patients under extracorporeal membrane oxygenation, moderate, moderate ARDS, severe, severe ARDS without ECMO, PBW, predicted body weight

Left panel of Fig. 4 shows the frequency distribution of patients according to lung recruitability between PEEP 5 and 15 cmH_2_O. The median amount of recruitable lung between PEEP 5 and 15 cmH_2_O was 6 [4-10]%, and was not significantly different between groups. PEEP_volume_ was significantly lower in ECMO patients, while ∆PEEP_5-15_-induced hyperinflation was significantly lower in the ECMO group and virtually inexistent (Fig. 2). A sensitivity analysis excluding the 3 patients with exclusion criteria yielded identical results (Additional file 4). The hyperinflation-to-recruitment ratio (a trade-off index of the adverse effects and benefits of PEEP increase from 5 to 15 cmH_2_O) was significantly lower in ECMO patients, as compared to both severe non-ECMO ARDS patients and moderate ARDS patients (Fig. 3, right panel). This ratio was lower than 1 in 8 (50%) moderate ARDS patients, 41 (69%) severe non-ECMO patients, and in 23 (96%) ECMO patients, i.e., virtually all ECMO patients responded to PEEP 15 cmH_2_O by recruited volume outweighing hyperinflation volume.

**Fig. 5:**
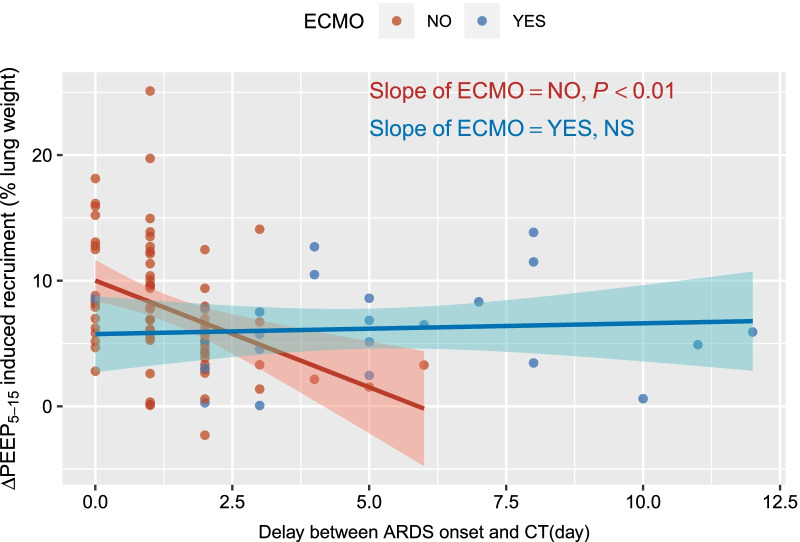
Relationship between ∆PEEP_5-15_-induced recruitment and delay between ARDS onset and CT, according to ECMO status. Each point is partial residuals of the multivariate model with ∆PEEP_5–15_-induced recruitment as the dependent variable, and the following independent variables: EELV at PEEP 5 cmH_2_O, poorly inflated lung at PEEP5 and the interaction between ECMO status × delay between CT and ARDS onset. Lines are regression lines according to ECMO status. Shadowed areas are 95% confidence interval for each regression line. ARDS, acute respiratory distress syndrome, CT, computed tomography, ECMO, extracorporeal membrane oxygenation, ∆PEEP_5-15_-induced recruitment, amount of recruitable lung between PEEP 5 and 15 cmH_2_O, EELV, end-expiratory lung volume, NS, non-statistically significant, PEEP, positive end-expiratory pressure

Multivariate analysis identified poorly inflated lung at PEEP5, EELV at PEEP5 and the interaction of ECMO status and delay between ARDS onset and CT as independent predictors of ∆PEEP_5-15_-induced recruitment (Additional file 5 and 6). ∆PEEP_5-15_-induced recruitment significantly decreased over time from ARDS onset in non-ECMO patients, but not in ECMO patients (Fig. 5).

### Tidal hyperinflation and tidal recruitment

The frequency distribution of patients according to tidal hyperinflation is reported in Fig. 4 (right panel). Tidal hyperinflation amounted to 0.3 [0.1–1.0] mL.kg^−1^ PBW. Tidal hyperinflation greater than 1 mL.kg^−1^ PBW was observed in 19 (25%) non-ECMO patients and 0 (0%) ECMO patients. As expected, tidal hyperinflation was significantly lower in ECMO patients, while tidal recruitment was significantly higher in non-ECMO severe ARDS patients (Fig. 2).

### Lung inhomogeneity

**Fig. 6:**
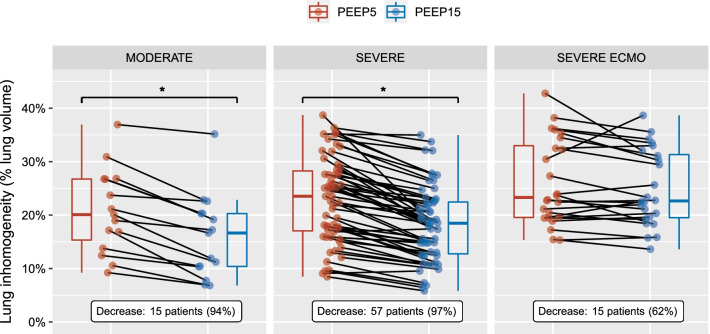
Lung inhomogeneities and PEEP. The extent of lung inhomogeneity of the individual patients with moderate ARDS (left panel), severe ARDS without ECMO (middle panel), and severe ARDS with ECMO (right panel) is reported at PEEP 5 (red circles) and 15 cmH_2_O (blue circles) * *p* < 0.05 between PEEP 5 and 15 cm H_2_O of the same category of severity. Lung inhomogeneities are registered to the following patent: WO 2013/088336, and agreement to use this patent for research purposes was obtained from the owner (Fondazione IRCCS Ca’ Granda Ospedale Maggiore Policlinico). ARDS, acute respiratory distress syndrome, ECMO, extracorporeal membrane oxygenation, MODERATE, moderate ARDS, PEEP, positive end-expiratory pressure, SEVERE, severe ARDS without ECMO, SEVERE ECMO, severe ARDS under ECMO.

The extent of lung inhomogeneity decreased significantly from PEEP 5 to PEEP 15 cmH_2_O in non-ECMO patients, but not in ECMO patients (Fig. 6), with substantial interindividual variations. Lung inhomogeneity extent decreased between PEEP5 and 15 cmH_2_O in 94% of moderate ARDS patients, 97% of severe ARDS patients, and 62% of severe ECMO patients.

### Compliance of the aerated lung at PEEP 5 cmH_2_O

**Fig. 7:**
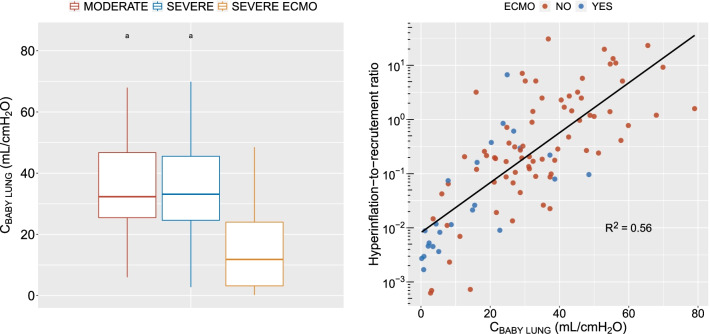
Compliance of the aerated lung at PEEP 5 cmH_2_O (C_BABY LUNG_). Left panel: C_BABY LUNG_ as a function of ARDS severity. Right panel. Relationship between hyperinflation-to-recruitment ratio and C_BABY LUNG_ as a function of ECMO status. Circles are individual datapoints. Black line is the regression line on the whole population. a, *p* < 0.05 vs severe ARDS under ECMO. ARDS, acute respiratory distress syndrome, ECMO, extracorporeal membrane oxygenation, MODERATE, moderate ARDS, PEEP, positive end-expiratory pressure, SEVERE, severe ARDS without ECMO, SEVERE ECMO, severe ARDS under ECMO

C_BABY LUNG_ was significantly lower in ECMO patients than in both severe ARDS patients without ECMO and moderate ARDS (Fig. 7, left panel). C_BABY LUNG_ was linearly related to the logarithm of the hyperinflation-to-recruitment ratio (Fig. 7, right panel).

Multivariate analysis identified BMI, ECMO, and EELV at PEEP 5 cmH_2_O as independent variables significantly associated with C_BABY LUNG_ (Additional file 7), while C_BABY LUNG_ was not independently associated with lung recruitability (Additional file 5 and 6).

## Discussion

The main findings of the study are the following: 1—lung recruitability between PEEP 5 and 15 cmH_2_O was not significantly different between categories of ARDS severity, 2—the trade-off between hyperinflation and recruitment induced by a PEEP change from 5 to 15 cmH_2_O was favorable in virtually all ECMO patients, 3—compliance of the aerated lung at PEEP 5 was significantly lower in ECMO patients, independently of lung recruitability, and may be a protective factor against PEEP-induced hyperinflation under ECMO.

Patient characteristics at inclusion were similar in the present study and in the largest French epidemiological study on critically ill adults with COVID-19 [27]. ECMO patients were older in the present study than in the ELSO [28] and the Paris–Sorbonne University Hospital Network [29] registries, but with similar BMI. However, PEEP level under ECMO was higher (15 vs 10 and 12 cmH_2_O, respectively), while VT, Δ*P*_rs_, and P_Plat,rs_ were substantially lower in the present study [28, 29]. In non-ECMO-patients, the amount of non-aerated lung at PEEP 5 cmH_2_O was similar in the present study and in 3 previous studies on COVID-19 patients under mechanical ventilation (approximately 40%) [5, 7, 8]. On the other hand, median lung recruitability between PEEP 5 and 15 cmH_2_O was substantially lower in the present study as compared to the study by Protti et al. (6% vs. 11% of lung weight) [8], with a relatively similar CT protocol and delay between ARDS onset and CT. In addition to differences between pre-intubation management and case-mix, this may be explained by CT acquisition at higher PEEP levels (10 and 20 cmH_2_O, respectively) in some obese patients in the latter study. In ECMO patients, the amount of non-aerated lung at PEEP 5 cmH_2_O was similar in our study and in non-COVID-19 ECMO patients (approximately 80%) [10].

An important finding of our study is that the delay from ARDS onset is an important confounding factor of lung recruitability and that lung recruitability decreases as early as during the first 3 days after ARDS onset. Surprisingly, this effect was not identified in ECMO patients and the following potential reasons may be hypothesized to explain this finding: patient selection for ECMO eligibility, lower sample size of the ECMO subgroup, beneficial impact of fluid administration required to maintain ECMO flow on lung recruitability, or preventive effect of ECMO on fibrotic changes potentially involved in the progressive loss of recruitability observed under standard ventilation [30], although this remains speculative.

To date, there is no undisputable threshold defining low or high recruiter patients with CT. Most studies defined high recruiters as patients with an amount of recruited lung between 5 and 45 cmH_2_O above the median value of the population [9, 10]. As approximately 50% of the recruitment between PEEP 5 and 45 cmH_2_O is achieved between PEEP 5 and 15 cmH_2_O [8, 9], and as the threshold defining high recruiter amounted to 10% of total lung weight in the seminal study by Gattinoni and coworkers [9], it can be speculated that the 6% median lung recruitment value identified in our study suggests that COVID-19 and non-COVID-19 ARDS are roughly similar regarding lung recruitability. Remarkably, recruitability was similar among categories of ARDS severity in our study, despite a stepwise increase in non-inflated lung compartment as severity increases, in striking contrast with Gattinoni’s study [9], suggesting that the underlying mechanisms responsible for lung consolidation are different between COVID-19 and non-COVID-19 ARDS. However, we failed to relate decreased compliance of the aerated lung to poor lung recruitability, suggesting that the mechanical characteristics of the aerated and non-aerated lung may be unrelated.

Another striking result of the present study is that the balance between hyperinflation and recruitment induced by a PEEP increase from 5 to 15 cmH_2_O is favorable in virtually all ECMO patients, as opposed to COVID-19 ARDS patients without ECMO. Interestingly, most of the patients included in large ECMO cohort studies on COVID-19 ARDS were ventilated with substantially lower PEEP levels [28, 29]. Our results suggests that the lower compliance of the aerated lung at PEEP 5 cmH_2_O during ECMO (a consequence of lower EELV) may prevent occurrence of PEEP-induced hyperinflation (Figs. 2 and 7). Hyperinflation may have nonetheless been undetected in ECMO patients as it may occur at a level below the resolution of CT, or since the decrease in CT density due to hyperinflation can be masked by the increased tissue mass in severe ARDS [31]. Hyperinflation was hence not detected during PEEP increase from 5 to 45 cmH_2_O in a previous study on ECMO patients [10]. Furthermore, we cannot exclude that overdistension without hyperinflation occurred at the interface between non-aerated and aerated lung units in some patients (see below) [19, 20]. Finally, the impact of reduced lung compliance on overdistension occurrence during PEEP increase in ECMO patients remains unknown.

A beneficial impact of PEEP increase on lung inhomogeneity extent was only identified in non-ECMO patients. As lung inhomogeneities act as stress raisers, this suggests that the energy load was more evenly distributed within the lung parenchyma at PEEP 15 in this subgroup of patients. This effect was not observed in ECMO patients, although with substantial interindividual variations, as was previously observed in non-COVID ARDS [20]. Moreover, PEEP increase was expected to be detrimental on stress raisers in 38% of ECMO patients, favoring individualization of PEEP settings.

Some limitations of the present study should be acknowledged. First, potentially recruitable lung at PEEP 45 cmH_2_O was not assessed, as this PEEP level was deemed excessive by our team [32] and others in COVID-19 ARDS [7] and higher PEEP levels may have led to different results. ECMO patients were studied a median of 4 days later than non-ECMO patients, and this delay may have impacted lung recruitability under ECMO, although this effect was ruled out by our multivariate analysis (Additional file 6, Fig. 5). Occurrence of complete airway closure [1] was not assessed in the present study, and this effect may have biased measurement of C_BABY LUNG_. Approximately 70% of the screened population (i.e., patients with COVID-19 pneumonia under invasive mechanical ventilation) lacked eligibility criteria, mostly because a CT was performed before intensive care admission, and the study population may be a biased subset of the initial population. Variable selection for multivariate analysis was performed on the basis of bivariable association, and this may have increased the risks of detecting spurious statistical associations [33]. Finally, the observational design of the study precludes any firm conclusion to be drawn regarding optimal ventilation settings in ARDS COVID-19 patients.

Nevertheless, the study has the following strengths. Lung evaluation was performed using CT, i.e., the reference method to perform a quantitative analysis of lung aeration and recruitment [15, 31, 34]. External validity of the study is expected to be good owing to the low rate of screen failure despite the context of pandemics, the substantial sample size for a CT study, and the multicenter design. Furthermore, the study is the first to assess lung recruitability in both ECMO and non-ECMO patients, in a homogeneous time frame (i.e., at the early phase of ARDS), thus minimizing potential confounding effects related to ventilator-induced lung injury or ventilator-associated pneumonia.

According to our results, a PEEP level around 13–15 cmH_2_O is required to overcome the superimposed hydrostatic pressure and prevent alveolar collapse in the majority of COVID-19 ARDS patients under ECMO. Higher PEEP levels may be required in patients with elevated chest wall to respiratory system elastance ratio, such as superobese patients, as part of this pressure level may be dissipated in the chest wall. In addition, a PEEP level of 15 cmH_2_O is associated with a favorable balance ratio between hyperinflation and recruitment induced by PEEP in virtually all COVID-19 ECMO patients. However, this PEEP level may be detrimental on stress raisers in a subset of ECMO patients favoring individualization of PEEP setting. Furthermore, the rationale for high PEEP use in patients under ECMO may be questionable as it relies on a putative protective effect on derecruitment related to the use of ultra-low tidal volume, while it may overdistend the lung, alter hemodynamics or increase the mechanical power transmitted to the lung from the ventilator. Finally, lung recruitability should be reassessed early in the course of COVID-19 ARDS without ECMO, as it decreases sharply over time.

## Conclusion

Lung recruitability of COVID-19 pneumonia is not significantly different among categories of ARDS severity, with substantial interindividual variations favoring individualization of PEEP setting. The balance between hyperinflation and recruitment induced by PEEP increase from 5 to 15 cmH_2_O appears to be favorable in virtually all ECMO patients, although lung inhomogeneities acting as stress raisers were not significantly improved by PEEP increase in this group of patients. A PEEP level of 12–15 cmH_2_O is required to counteract compressive forces leading to lung collapse in most ECMO patients. In non-ECMO patients, lung recruitability decreases steadily during the first 3 days after ARDS onset and justifies reevaluation of the PEEP setting on a daily basis.

## Supplementary Information

**Additional file 1: **Computation of compliance of the already aerated lung at PEEP 5 (C_BABY LUNG_)

**Additional file 2: **Flow chart of the study

**Additional file 3: **Missing data per variable

**Additional file 4: **Sensitivity analysis

**Additional file 5: **Univariate analysis of variables associated with lung recruitment

**Additional file 6: **Multivariate analysis of variables associated with PEEP-induced lung recruitment

**Additional file 7: **Univariate and multivariate analyses of variables associated with C_BABY LUNG_

## Data Availability

The datasets used and/or analyzed during the current study are available from the corresponding author on reasonable request.

## Abbreviations

ARDS: Acute respiratory distress syndrome
BMI: Body mass index
C_BABY LUNG_: Compliance of the aerated lung at PEEP 5 cmH_2_O, corrected for PEEP-induced recruited aerated volume
CT: Computed tomography
CT_Expi-Inspi_: CT acquired during end-expiratory and end-inspiratory pauses at the PEEP level set by the clinician
CT_PEEP5-15_: CT acquired during end-expiratory pauses at PEEP 5 and 15 cmH_2_O
∆PEEP_5-15_: Change in PEEP from 5 to 15 cmH_2_O
Δ*P*_rs_: Airway driving pressure
ECMO: Extracorporeal membrane oxygenation
EELV: End-expiratory aerated lung volume
FiO_2_: Inspired oxygen fraction
HU: Hounsfield units
PaO_2_/FiO_2_: Ratio of oxygen arterial partial pressure to inspired oxygen fraction
PBW: Predicted body weight
PEEP: Positive end-expiratory pressure
PEEP_tot,rs_: Total PEEP of the respiratory system
PEEP_volume_: Change in lung aerated volume induced by PEEP change from 5 to 15 cmH_2_O
P_Plat,rs_: Plateau pressure of the respiratory system
Rec_Aer vol_: Recruited aerated volume between PEEP5 and 15 cmH_2_O
VT: Tidal volume
VT_CT_: Tidal volume was assessed on CT
