# Supplementary material for: Response to PEEP in COVID-19 ARDS patients with and without extracorporeal membrane oxygenation. A multicenter case–control computed tomography study
Source: Crit Care. 2022 Jul 2;26:195. doi: 10.1186/s13054-022-04076-z (PMC9250720; doi:10.1186/s13054-022-04076-z)
Supplement: Supplementary file 1 — Additional file 1: Computation of compliance of the already aerated lung at PEEP 5 (CBABY LUNG) [file 13054_2022_4076_MOESM1_ESM.docx]

**Additional file 1. Computation of** **compliance of the already aerated lung at PEEP 5 (C_BABY LUNG_)**


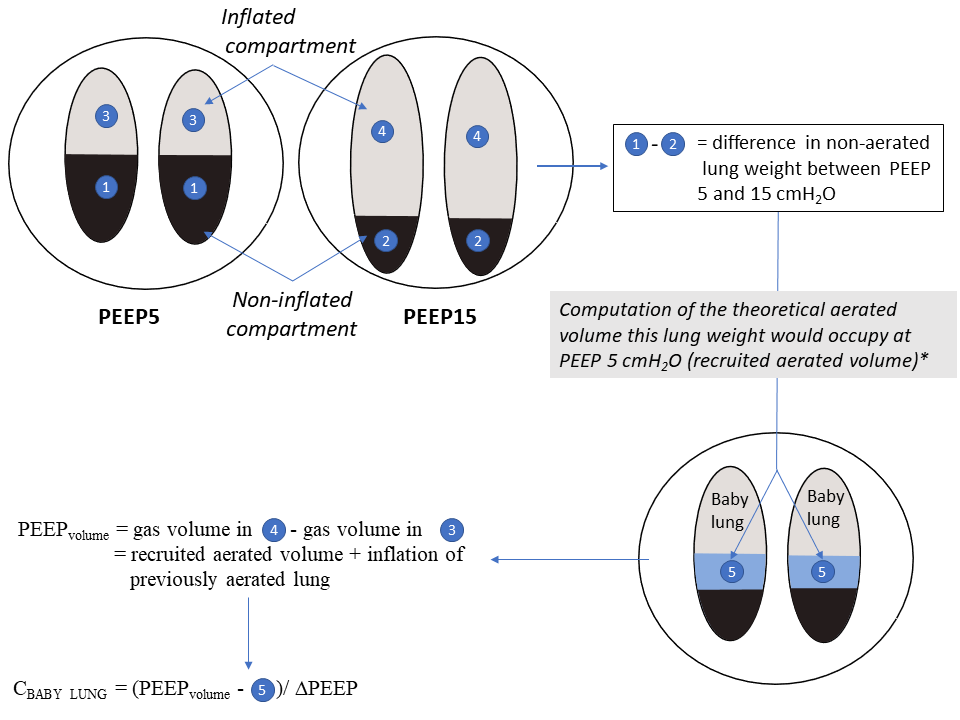


Baby lung compliance (C_BABY LUNG_) refers to the elastic properties of the already aerated lung at PEEP 5 cmH_2_O. Classical computation of compliance between PEEP 5 and 15 cmH_2_O (i.e., change in lung aerated volume divided by change in PEEP) overestimates C_BABY LUNG_ as recruited alveoli account partly for the change in aeration.

As recruitment assessed by computed tomography is computed as the difference in non-aerated lung weight between PEEP levels, a computation of recruited aerated volume (Rec_Aer vol_) from recruited lung weight was performed using the methodology proposed by Paula and coworkers*, assuming that recruitable alveoli would remain aerated at PEEP 5 cmH_2_O and have equilibrated to a level of expansion equivalent to that of other already open alveoli at PEEP 5.

PEEP_volume_ (change in lung aerated volume induced by PEEP increase from 5 to 15 cmH_2_O), was computed as the difference in the total volume of gas within the lungs between PEEP 15 and 5 cmH_2_O.

C_BABY LUNG_ was finally computed as (PEEP_volume_ - Rec_Aer vol_)/∆PEEP (i.e. 10 cmH_2_O)

* Paula LFS da C, Wellman TJ, Winkler T, et al. Regional Tidal Lung Strain in Mechanically Ventilated Normal Lungs. *J Appl Physiol*. 2016;121(6):1335–1347).

PEEP, positive-end expiratory pressure
