# Supplementary material for: Response to PEEP in COVID-19 ARDS patients with and without extracorporeal membrane oxygenation. A multicenter case–control computed tomography study
Source: Crit Care. 2022 Jul 2;26:195. doi: 10.1186/s13054-022-04076-z (PMC9250720; doi:10.1186/s13054-022-04076-z)
Supplement: Supplementary file 2 — Additional file 2: Flow chart of the study [file 13054_2022_4076_MOESM2_ESM.docx]

**Additional file 2. Flow chart of the study**


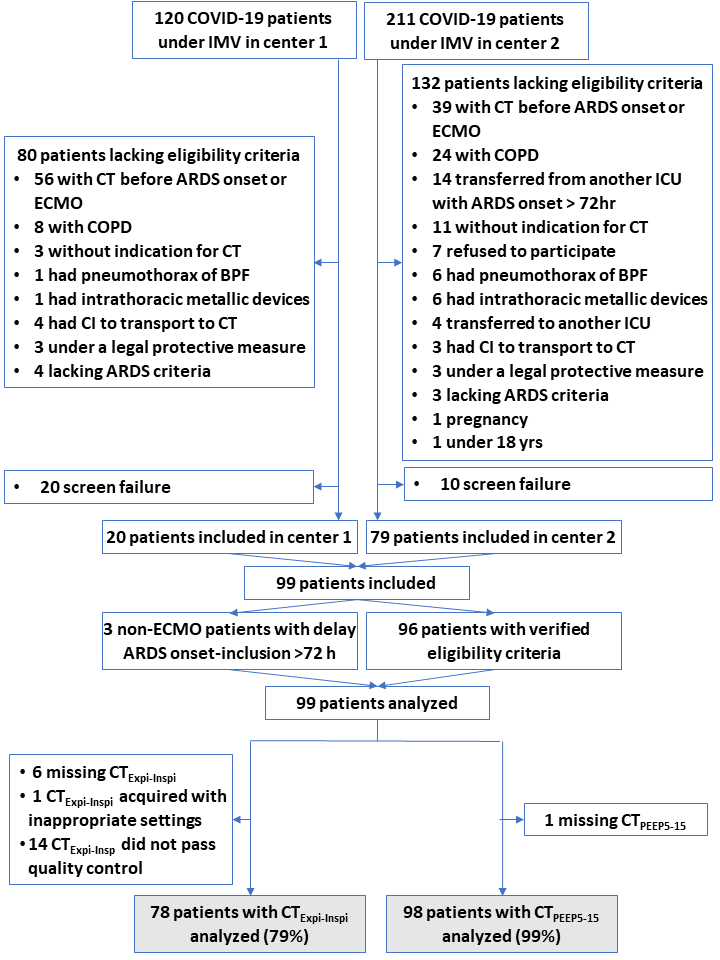


ARDS, acute respiratory distress syndrome; BPF, broncho-pleural fistula; CI, contra-indication; COPD, chronic obstructive pulmonary disease; CT, computed tomography; CT_Expi-Inspi_, couple of computed tomography image volumes acquired at end-expiration and at end-inspiration; CT_PEEP5-15_, couple of computed tomography image volumes acquired at PEEP5 and PEEP15 cmH_2_O; ECMO, extracorporeal membrane oxygenation; ICU, intensive care unit; IMV, invasive mechanical ventilation; PEEP, positive end-expiratory pressure.
