# Supplementary material for: Response to PEEP in COVID-19 ARDS patients with and without extracorporeal membrane oxygenation. A multicenter case–control computed tomography study
Source: Crit Care. 2022 Jul 2;26:195. doi: 10.1186/s13054-022-04076-z (PMC9250720; doi:10.1186/s13054-022-04076-z)
Supplement: Supplementary file 3 — Additional file 3: Missing data per variable [file 13054_2022_4076_MOESM3_ESM.docx]

**Additional file 3. Missing data per variable**

| Variables | Number of missing data (%) |
| --- | --- |
| Demographic variables | |
| Sex | 0 (0%) |
| Age | 0 (0%) |
| BMI | 0 (0%) |
| Delay between hospital admission and CT | 0 (0%) |
| Delay between ICU admission and CT | 0 (0%) |
| Delay between ARDS onset and CT | 0 (0%) |
| Delay between IMV onset and CT | 0 (0%) |
| SAPS 2 at ICU admission | 0 (0%) |
| SOFA score at inclusion | 0 (0%) |
| Prone position in the 24h preceding CT | 0 (0%) |
| iNO in the 24h preceding CT | 0 (0%) |
| NMBA in the 24h preceding CT | 0 (0%) |
| RRT in the 24h preceding CT | 0 (0%) |
| Inotropes in the 24h preceding CT | 0 (0%) |
| Vasopressor in the 24h preceding CT | 0 (0%) |
| Physiological variables |  |
| PEEP | 0 (0%) |
| VT | 0 (0%) |
| RR | 0 (0%) |
| PEEP_tot,rs_ | 0 (0%) |
| P_plat,rs_ | 0 (0%) |
| P_peak_ | 1 (1%) |
| ΔP_rs_ | 0 (0%) |
| E_rs_ | 0 (0%) |
| pH | 0 (0%) |
| PaO_2_ | 0 (0%) |
| FiO_2_ or FmO_2_ | 0 (0%) |
| PaCO_2_ | 0 (0%) |
| Bicarbonates | 0 (0%) |
| Lactate | 0 (0%) |
| CT variables |  |
| Lung weight | 0 (0%) |
| Non-inflated lung at PEEP 5 | 1 (1%) |
| EELV at PEEP 5 | 1 (1%) |
| ∆PEEP_5-15_ induced recruitment | 1 (1%) |
| PEEP_volume_ | 1 (1%) |
| ∆PEEP_5-15_ induced hyperinflation | 1 (1%) |
| Hyperinflation to recruitment ratio | 1 (1%) |
| Total superimposed pressure | 1 (1%) |
| Tidal recruitment | 21 (21%) |
| Tidal hyperinflation | 21 (21%) |
| C_BABY LUNG_ | 1 (1%) |
| Outcome variables |  |
| ICU mortality | 0 (0%) |
| Day-90 mortality | 0 (0%) |
| Ventilator-free days at day-90 | 1 (1%) |
| ICU length of stay | 0 (0%) |
| Hospital length of stay | 1 (1%) |

ARDS, acute respiratory distress syndrome; BMI, body mass index; C_BABY LUNG_, compliance of the aerated lung between PEEP 5 and 15 cmH_2_O corrected for PEEP-induced recruitment corrected for PEEP-induced recruitment; CT, computed tomography; ∆PEEP_5-15_ induced hyperinflation, hyperinflation induced by PEEP change from 5 to 15 cmH_2_O; ∆PEEP_5-15_ induced recruitment, recruitment induced by PEEP change from 5 to 15 cmH_2_O; PEEP_volume_, change in lung aeration induced by PEEP increase from 5 to 15 cmH_2_O; ΔP_rs_, driving pressure of the respiratory system; ECMO, extracorporeal membrane oxygenation; EELV, end-expiratory lung volume; E_rs_, elastance of the respiratory system; FiO_2_, inspired fraction of oxygen; FmO_2_, ECMO membrane oxygen fraction; ICU, intensive care unit; IMV, invasive mechanical ventilation; iNO, inhaled nitric oxide; NMBA, neuromuscular blocking agents; PaCO_2_, carbo dioxide partial pressure in arterial blood; PaO_2_, oxygen partial pressure in arterial blood; PBW, predicted body weight; PEEP, positive end-expiratory pressure; PEEP_tot,rs_, total PEEP of the respiratory system; P_peak_, peak airway pressure; P_plat,rs_, plateau pressure of the respiratory system; RR, respiratory rate; RRT, renal replacement therapy; SAPS2, simplified acute physiology score; VT, tidal volume.
