# Supplementary material for: Response to PEEP in COVID-19 ARDS patients with and without extracorporeal membrane oxygenation. A multicenter case–control computed tomography study
Source: Crit Care. 2022 Jul 2;26:195. doi: 10.1186/s13054-022-04076-z (PMC9250720; doi:10.1186/s13054-022-04076-z)
Supplement: Supplementary file 4 — Additional file 4: Sensitivity analysis [file 13054_2022_4076_MOESM4_ESM.docx]

**Additional file 4. Sensitivity analysis excluding the 3 patients with exclusion criteria (ARDS onset > 72h) erroneously included in the study**


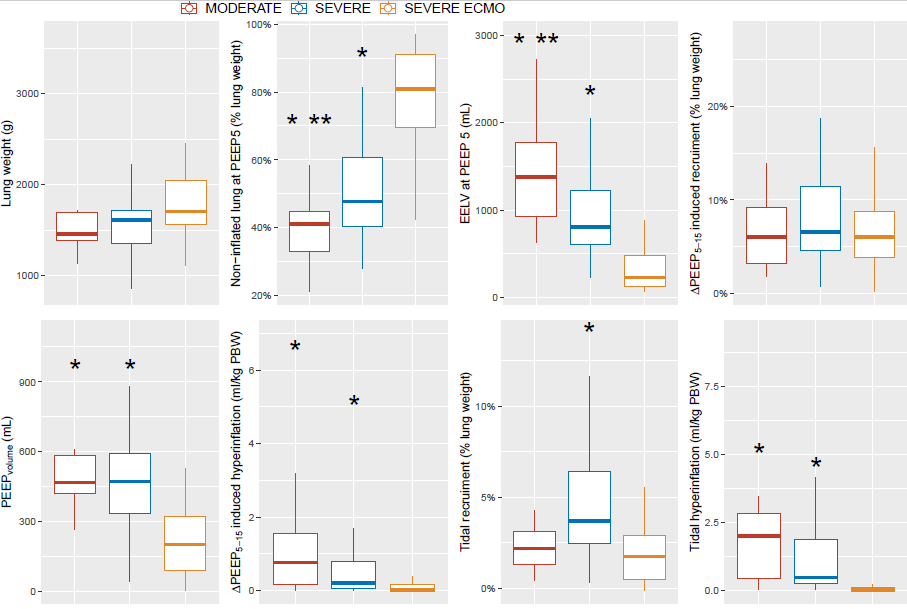


*, p<0.05 vs severe ARDS with ECMO; **, p<0.05 vs severe ARDS without ECMO.

ARDS, acute respiratory distress syndrome; ∆_PEEP5-15_, change in PEEP from 5 to 15 cmH_2_O; ECMO, extracorporeal membrane oxygenation; EELV, end-expiratory lung volume; PBW, predicted body weight; PEEP, positive-end-expiratory pressure; PEEP_volume_, change in lung aeration induced by PEEP change from 5 to 15 cmH_2_O.
