# Supplementary material for: Response to PEEP in COVID-19 ARDS patients with and without extracorporeal membrane oxygenation. A multicenter case–control computed tomography study
Source: Crit Care. 2022 Jul 2;26:195. doi: 10.1186/s13054-022-04076-z (PMC9250720; doi:10.1186/s13054-022-04076-z)
Supplement: Supplementary file 5 — Additional file 5: Univariate analysis of variables associated with lung recruitment [file 13054_2022_4076_MOESM5_ESM.docx]

**Additional file 5. Univariate analysis of variables associated with lung recruitment (expressed in percentage of lung weight)**

| Variables | Univariate  slope ± SE | Univariate p-value |
| --- | --- | --- |
| Sex male | - | 0.62 |
| Age (per 10-year increase) | -0.1±0.0 | **0.12** |
| BMI | - | 0.63 |
| Delay between hospital admission and CT | - | 0.62 |
| Delay between ICU admission and CT | - | 0.20 |
| Delay between CT and ARDS onset (per 1-day increase) | -0.3±0.2 | **0.14** |
| SAPS2 | - | 0.55 |
| ARDS severity | - | 0.64 |
| ECMO | -1.3±1.2 | 0.30 |
| PEEP | - | 0.76 |
| PEEP_tot,rs_ | - | 0.87 |
| VT | - | 0.22 |
| P_plat,rs_ | - | 0.31 |
| ΔP_rs_ | - | 0.34 |
| E_rs_ | - | 0.42 |
| FiO_2_ (per 0.1 increase) | 0.4±0.2 | **0.06** |
| pH | - | 0.49 |
| PaO_2_ | - | 0.62 |
| PaCO_2_ | - | 0.83 |
| Lung weight | - | 0.85 |
| Non-inflated lung at PEEP5 | - | 0.76 |
| Poorly-inflated lung at PEEP5 (per 10% of lung weight increase) | 1.0±0.4 | **<0.05** |
| Normally-inflated lung at PEEP5 (per 10% of lung weight increase) | -1.4±0.5 | **<0.01** |
| Hyperinflated lung at PEEP 5 (per 1-ml.kg^-1^ PBW increase) | -0.5±0.2 | **<0.05** |
| EELV at PEEP5 (per 100-mL increase) | -0.2±0.1 | **<0.05** |
| Total superimposed pressure at PEEP5 | - | 0.27 |
| C_BABY LUNG_ | - | 0.77 |

Bold values are p values below 0.2 candidate for inclusion in the multivariate model.

ARDS, acute respiratory distress syndrome; BMI, body mass index; C_BABY LUNG_, compliance of the aerated lung between PEEP 5 and 15 corrected for PEEP-induced recruitment; CT, computed tomography; ΔP_rs_, driving pressure of the respiratory system; ECMO, extracorporeal membrane oxygenation; EELV, end-expiratory lung volume; E_rs_, elastance of the respiratory system; FiO_2_, inspired oxygen fraction; ICU, intensive care unit; PaCO_2_, carbon dioxide partial pressure in arterial blood; PaO_2_, oxygen partial pressure in arterial blood; PBW, predicted body weight; PEEP, positive end-expiratory pressure; PEEP_tot,rs_, total PEEP of the respiratory system; P_plat,rs_, plateau pressure of the respiratory system; SAPS2, simplified acute physiology score-2; SE, standard error; VT, tidal volume.
