# Supplementary material for: Response to PEEP in COVID-19 ARDS patients with and without extracorporeal membrane oxygenation. A multicenter case–control computed tomography study
Source: Crit Care. 2022 Jul 2;26:195. doi: 10.1186/s13054-022-04076-z (PMC9250720; doi:10.1186/s13054-022-04076-z)
Supplement: Supplementary file 6 — Additional file 6: Multivariate analysis of variables associated with PEEP-induced lung recruitment [file 13054_2022_4076_MOESM6_ESM.docx]

**Additional file 6. Multivariate analysis of variables associated with ∆PEEP_5-15_ induced lung recruitment (expressed in percentage of lung weight)**

| Variables | Multivariate  slope ± SE | Multivariate p-value |
| --- | --- | --- |
| Poorly-inflated lung at PEEP5 (per 10% of lung weight increase) | 1.3±0.5 | <0.01 |
| EELV at PEEP5 (per 100-mL increase) | -0.3±0.1 | <0.001 |
| Delay between CT and ARDS onset × ECMO interaction |  | <0.01 |
| - without ECMO (per 1-day increase) | -1.7±0.5 | <0.01 |
| - with ECMO (per 1-day increase) | 0.1±0.3 | NS |

ARDS, acute respiratory distress syndrome; CT, computed tomography; ∆PEEP_5-15_, change in PEEP from 5 to 15 cm H_2_O; ECMO, extracorporeal membrane oxygenation; EELV, end-expiratory lung volume; NS, not significant; PEEP, positive end-expiratory pressure; SE, standard error.

Variables with p<0.2 in univariate analysis were included in the full model (i.e., age, delay between CT and ARDS onset, FiO_2_, EELV at PEEP5, poorly-inflated lung at PEEP5 and hyperinflated lung at PEEP 5). ECMO was forced into the multivariate model as a significant interaction with delay between CT and ARDS onset was identified. Normally-inflated lung at PEEP5 was not included in the full model for collinearity with EELV at PEEP5.

Adjusted R^2^ of multivariate model: 0.24.
