# Supplementary material for: Response to PEEP in COVID-19 ARDS patients with and without extracorporeal membrane oxygenation. A multicenter case–control computed tomography study
Source: Crit Care. 2022 Jul 2;26:195. doi: 10.1186/s13054-022-04076-z (PMC9250720; doi:10.1186/s13054-022-04076-z)
Supplement: Supplementary file 7 — Additional file 7: Univariate and multivariate analyses of variables associated with CBABY LUNG [file 13054_2022_4076_MOESM7_ESM.docx]

**Additional file 7. Univariate and multivariate analyses of variables associated with C_BABY LUNG_**

| Variables | Univariate slope±SE | Univariate p-value | Multivariate slope ±SE | Multivariate p-value |
| --- | --- | --- | --- | --- |
| Sex male (ref=Female) | 10.2±4.2 | <0.05 | - | NS |
| Age (per 10-yr increase) | 6.3±1.4 | <0.001 | - | NS |
| BMI (per 1 kg.m^-2^increase) | -1.1±0.2 | <0.001 | -0.4±0.2 | <0.05 |
| Delay between ARDS onset and CT (per 1-day increase) | -1.7±0.7 | <0.05 | - | NS |
| SAPS2 | - | 0.85 | - | - |
| Severe ARDS (ref=moderate) | -7.8±5.1 | 0.13 | - | NS |
| ECMO (ref=No) | -19.1±3.8 | <0.001 | -7.2 ± 3.3 | <0.05 |
| Lung weight (g) | - | 0.36 | - | - |
| EELV at PEEP5 (per 100-mL increase) | 2.0±0.2 | <0.001 | 1.5±0.2 | <0.001 |

ARDS, acute respiratory distress syndrome; BMI, body mass index; C_BABY LUNG_, compliance of the aerated lung between PEEP 5 and 15 corrected for PEEP-induced recruitment; CT, computed tomography; ECMO, extracorporeal membrane oxygenation; EELV, end-expiratory lung volume; NS, not statistically significant; PEEP, positive end-expiratory pressure; SAPS2, simplified acute physiology score-2; SE, standard error.

Variables with p<0.2 in univariate analysis were included in the full model. The interaction between ECMO and delay between ARDS onset and CT was not statistically significant. Collinearity between variables was checked and ruled out.

Adjusted R^2^ of the multivariate model : 0.56.
